# Effect of boiling water soaking on the mechanical properties and durability of nanoclay-enhanced bamboo and glass fiber epoxy composites

**DOI:** 10.1038/s41598-025-87912-w

**Published:** 2025-01-29

**Authors:** Syed Mansoor Ahmad, M. C. Gowrishankar, Manjunath Shettar

**Affiliations:** Department of Mechanical and Industrial Engineering, Manipal Institute of Technology, Manipal Academy of Higher Education, Manipal, 576104 India

**Keywords:** Bamboo fiber, Nanoclay, Hybridization, Boiling water-soaking, Mechanical properties, Materials science, Mechanical engineering

## Abstract

Fiber-reinforced polymer composites are subjected to harsh environmental conditions over the course of their designed lifespan. Studying the aging process of fiber-reinforced polymer composites exposed to boiling water is critical for improving their durability. This study uses a hand lay-up technique to fabricate composites from glass fiber, bamboo fiber, nanoclay, and epoxy. Tensile and flexural tests are conducted following ASTM standards. The % of water uptake of the boiling water-soaked specimens is measured, and the performance of the dry composites is compared with those of boiling water-soaked composites. The results show that boiling water-soaking conditions have an adverse impact on all the composites. Boiling water-soaked epoxy samples show a reduction in tensile properties by 25 and 30% and flexural properties by 18 and 22% under processes 1 and 2 of boiling water soaking, respectively, compared to dry ones. Meanwhile, for fiber-reinforced composites, the tensile properties decrease by 19 and 27%, and the flexural properties decline by 12 and 20% under processes 1 and 2 of boiling soaking, respectively. However, incorporating nanoclay enhances the tensile and flexural properties of the epoxy and the composites by 5 to 7% and 10 to 12%, respectively. The water absorption rate and the impact of boiling water-soaking on composite strength decrease with the addition of nanoclay. Additionally, nanoclay reduces the percentage of reduction in tensile properties by 17 and 26% and in flexural properties by 11 and 18% under processes 1 and 2 of boiling soaking, respectively. SEM analysis of the fracture surfaces reveals the causes of specimen failure under tensile load, with distinct differences between dry and boiling water-soaked specimens.

##  Introduction

Fiber-reinforced polymer composites are increasingly becoming a part of daily life due to their numerous advantages, allowing them to compete with traditional materials like metals and alloys. These composites are known for their lightweight nature, rigidity, strength, and excellent fatigue resistance. They have proven their value initially developed for high-performance industries such as transportation (air, sea, rail), medical devices, civil engineering, automobiles, and sports^1,2^. Among their various benefits, using natural fibers, particularly plant-based fibers, to reinforce composite materials offers notable advantages. These fibers are often more cost-effective than synthetic ones. Since natural fibers are renewable and biodegradable, their integration into composites reduces environmental impact compared to conventional composites^3^. Various natural fibers, including flax, alfa, kenaf, hemp, jute, and bamboo, are being utilized in composite manufacturing, presenting an attractive alternative that is gaining popularity^4^. However, these materials are sensitive to environmental changes, are hydrophilic, and exhibit low resistance to moisture absorption, which poses a significant challenge^5^. Addressing and managing the issue of moisture sorption by natural fibers is crucial for advancing these composite materials.

Understanding the effect of boiling water soaking on polymer composites is essential because it helps assess the material’s durability, performance, and suitability for various applications, particularly in environments where exposure to moisture and elevated temperatures is expected. By studying the effects of boiling water soaking, engineers can estimate the composite’s service life in humid or wet conditions, ensuring the material’s reliability in real-world applications like automotive, marine, and construction sectors. To counter the effects of boiling water soaking on natural fiber-reinforced polymer composites, researchers have used many strategies to improve moisture resistance, mechanical durability, and long-term performance^6^. These strategies primarily target reducing water absorption by modifying the fibers, matrix, or the interface between them. The different strategies are surface modification of natural fibers^7^, modifying the polymer matrix with nano-additives^8^, and combining natural fibers with synthetic fibers or using hybrid composites^9^. Combining these strategies enables the development of materials that retain their mechanical integrity, reduce moisture-related degradation, and extend their applicability to various industries, including automotive, construction, and marine sectors.

Binti Mohd Hafidz et al.^10^ studied how treating kenaf fibers with NaOH impacts the water absorption of kenaf fiber-unsaturated polyester composites. Both untreated and treated with NaOH fibers are used to prepare the composites. The findings indicate that NaOH treatment significantly decreases the moisture uptake in the treated kenaf fiber composites compared to untreated kenaf fiber composites by enhancing interfacial bonding. SEM analysis confirms that NaOH treatment improves the composite’s interfacial bonding and mechanical properties. Melkamu et al.^11^ investigated the effect of alkaline-surface treatment on the water absorption properties of sisal fiber-reinforced polyester composites. The water absorption of 30 wt% untreated sisal fiber-reinforced polyester composites is 10%. In contrast, the water absorption of treated sisal fiber-reinforced polyester composites is just 2.8%. Gunti et al.^12^ studied the water absorption effect on untreated and treated elephant grass fiber-PLA composites. A water absorption of 11.17% is observed in untreated elephant grass fiber-PLA composites, whereas a water absorption of 6% is observed in alkali-treated elephant grass fiber-PLA composites.

Chee et al.^13^ studied the influence of nanoclay on moisture absorption of bamboo-kenaf-epoxy hybrid composites. Adding nanoclay reduces the moisture absorption of composites from 11 to 5%. Ramakrishnan et al.^14^ studied the effect of nanoclay addition on the water absorption behaviour of jute fiber-reinforced epoxy composites. Adding 1 and 3 wt% of nanoclay decreases water absorption (%) by 9 and 20%, respectively. Nanoclay facilitates better interfacial adhesion between fibers and epoxy, forming an intercalated structure that renders better moisture barrier properties by delaying moisture absorption.

Ng et al.^15^ studied pineapple leaf-glass fiber polypropylene hybrid composites’ mechanical properties and water uptake behaviors. Results reveal that combining pineapple leaf fibers with glass fibers improves the composite laminates’ mechanical properties and moisture uptake resistance. The hybrid composite laminates absorb less moisture (8.09%) than the pineapple leaf fiber laminates (11.66%) because of the addition of glass fibers. Glass fibers are hydrophobic, making them moisture-resistant. Zhang et al.^16^ investigated the effect of hybridization on water absorption properties of bamboo/glass-reinforced polybenzoxazine hybrid composite. Treated bamboo fiber-reinforced composites display a moisture uptake of 3.64% and 5.16%, and bamboo/glass-reinforced hybrid composites display a moisture uptake of 3.11% and 4.26%, under 25 °C and 80 °C water immersion, respectively. O˘GUZ et al.^17^ and Ozbek et al.^18^ investigated the hydrothermal aging effect on basalt and glass/basalt hybrid composite pipes. Hybridizing glass/basalt declines the water gain ratio by 45 to 80% and 50 to 74% under distilled and sea water soaking, respectively, compared to basalt composites.

The combined effects of glass fibers, bamboo fibers, and nanoclay on the mechanical properties and water uptake properties of epoxy-based composites are not extensively covered in the literature. In addition, studying the aging process of fiber-reinforced polymer composites exposed to boiling water is critical for improving their durability, which is unexplored as per the literature. This area holds significant potential for broadening the applications of fiber-reinforced polymer composites, particularly in the construction, marine, and automotive sectors. This study examines the influence of boiling water immersion on the mechanical properties of bamboo, glass fiber, and hybrid epoxy-based composites. It also compares the tensile and flexural properties of dry and boiling water-immersed composites. Furthermore, the study explores how nanoclay affects these composites’ resistance to boiling water soaking and mechanical properties. Fracture surfaces of dry and water-soaked specimens under tensile stress are analyzed to determine the causes of failure.

##  Methodology

### Materials

Epoxy resin (L-12) and hardener (K-6), with a mixing ratio of 10:1, are procured from Atul Polymers, Gujarat, India. Glass fiber reel with a bi-directional orientation is procured from Yuje Enterprises, Bengaluru, India. A surface-modified nanoclay is procured from Sigma Aldrich. The bi-directional-woven bamboo fiber reel is procured from Shreenath Weaving Industries in Gandhinagar, Gujarat, India. Some of the properties of selected materials for the work are presented in Table 1.

**Table 1:** Properties of selected material.

Sl No	Material Name	Properties
1	Glass fiber	Tensile strength (MPa) − 1720–1950 Tensile modulus (GPa) − 72–76 Density (g/cm^3^) − 2.5
2	Epoxy resin (L-12) and hardener (K-6)	Tensile strength (MPa) − 55–70 Tensile modulus (GPa) − 2.5–04 Flexural strength (MPa) − 120–140 Density (g/cm^3^) − 1.15
3	Nanoclay (Montmorillonite (MMT))	Appearance (color) - White to off-white Appearance (form) - Powder Size - < 20 μm Bulk density − 200–500 kg/m^3^ Surface modified contains 15–35 wt% octadecylamine, 0.5–5 wt% aminopropyltriethoxysilane.
4	Bamboo fiber	Tensile strength (MPa) − 540–630 Tensile modulus (GPa) − 11–17 Density (g/cm^3^) − 0.8

### Composites preparation

**Fig. 1:**
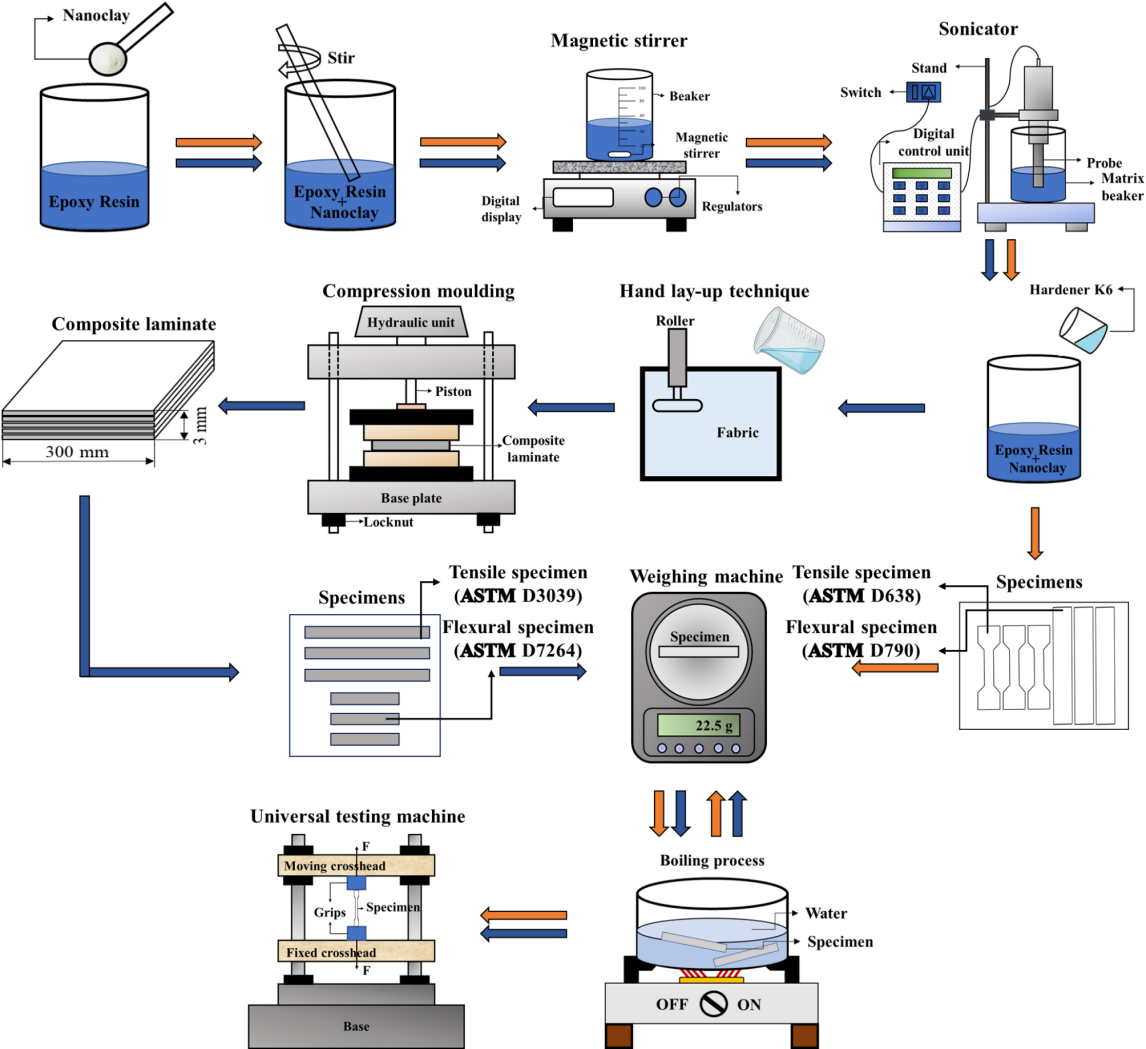
Preparation and testing of composites.

The fabrication of epoxy and epoxy-nanoclay composite samples is carried out using a conventional casting technique. Furthermore, bamboo/glass fiber epoxy and hybrid composite laminates are produced using the hand lay-up method, followed by compression molding at 100 bar and 50℃ for a period of 24 h. The specific procedures for laminate preparation are detailed in Fig. 1. Two sets of laminates are fabricated, with and without nanoclay, as specified in Tables 2 and 3. The notations E, BF, GF, and NF refer to epoxy, bamboo fiber, glass fiber, and nanoclay filler, respectively.

The process begins with incorporating nanoclay into the matrix material to achieve a uniform nanoclay dispersion throughout the matrix. Subsequently, magnetic stirring at 500 rpm for 30 min and sonication for 15 min are employed to break down agglomerates and ensure a homogeneous distribution of the nanoclay particles within the matrix. NaOH-treated bamboo fibers are employed to fabricate the composites. The detailed procedure for treating bamboo fiber is presented in our previous work^19^. For 50BF50E composites, 6 layers of bamboo fiber mat are used; for 50GF50E composites, 10 layers of glass fiber mat are used. Meanwhile, for 25BF25GF50E composites, 3 layers of bamboo and 5 layers of glass fiber mat are used. The specimens are cut from prepared laminates as per ASTM standards. The specimens are dipped in boiling water (Tap water), and their weight is observed daily to assess the water uptake percentage. This study thoroughly compares the mechanical properties of dry specimens with those subjected to boiling water immersion for all the prepared composites.

**Table 2:** Composite material’s composition (without nanoclay) and notations.

Bamboo Fiber wt%	Glass Fiber wt%	Epoxy wt%	Notation
-	-	100	100E
50	-	50	50BF50E
25	25	50	25BF25GF50E
-	50	50	50GF50E

**Table 3:** Composite material’s composition (with nanoclay) and notations.

Bamboo Fiber wt%	Glass Fiber wt%	Epoxy wt%	Nanoclay Filler wt%	Notation
-	-	98	2	98E2NF
50	-	48	2	50BF48E2NF
25	25	48	2	25BF25GF48E2NF
-	50	48	2	50GF48E2NF

### Mechanical characterization of composites

#### Tensile test

The ZWICK ROELL automated Universal Testing Machine (UTM) is employed in the tensile testing of nanoclay-epoxy and fiber-reinforced epoxy composite materials. The tests follow ASTM D638, which is concerned with plastics’ tensile properties, and ASTM D3039 for fiber-reinforced polymers. This ensures that testing covers those two types of material comprehensively and aligns with the set ways to get dependable results. The speed of the jaws is 1 mm/min.

#### SEM analysis

After tensile testing, SEM analysis is performed on the specimen using a ZEISS Scanning Electron Microscope (SEM). The sample is cut to size and securely mounted on the microscope. Before performing SEM analysis, a small sputter coater is used to apply a tinny layer of conductive substance on the specimens. The coating procedure takes 10 min to complete.

#### Flexural test

The ZWICK ROELL automated Universal Testing Machine (UTM) is utilized to perform a Three-point flexural test on composite, following ASTM D790 standards for 100E and 98E2NF specimens, as well as ASTM D7264 for fiber-reinforced composites. The speed of the jaws is 2 mm/min.

#### Boiling water soaking

The procedure is conducted as per ASTM D5229 standard (Procedure BBFF) in two ways (as presented in Table 4) to compare the results. Process 1 is the soaking of specimens in boiling water (tap water), which implies that samples are boiled in water for two hours and then dried (kept outside) for 22 h. Process 2 is to keep the specimens in the boiling water for 2 h and leave the specimens in the water for 22 h. These processes are repeated each day for 10 days, leading to a total boiling time of 20 h.

The water uptake percentage is determined by:

$$\% {\text{ of water uptake }}=\frac{{({\text{Weight after absorption}}-{\text{Weight of dry specimen}}) \times 100}}{{{\text{Weight of dry specimen}}}}$$

**Table 4:** Boiling soaking processes.

	Explanation
Process 1	Specimens are boiled for 2 h in water and dried (kept outside) for 22 h.
Process 2	Specimens are boiled for 2 h in water and kept in water for 22 h.

## Results and discussion

###  Water uptake curves

**Fig. 2:**
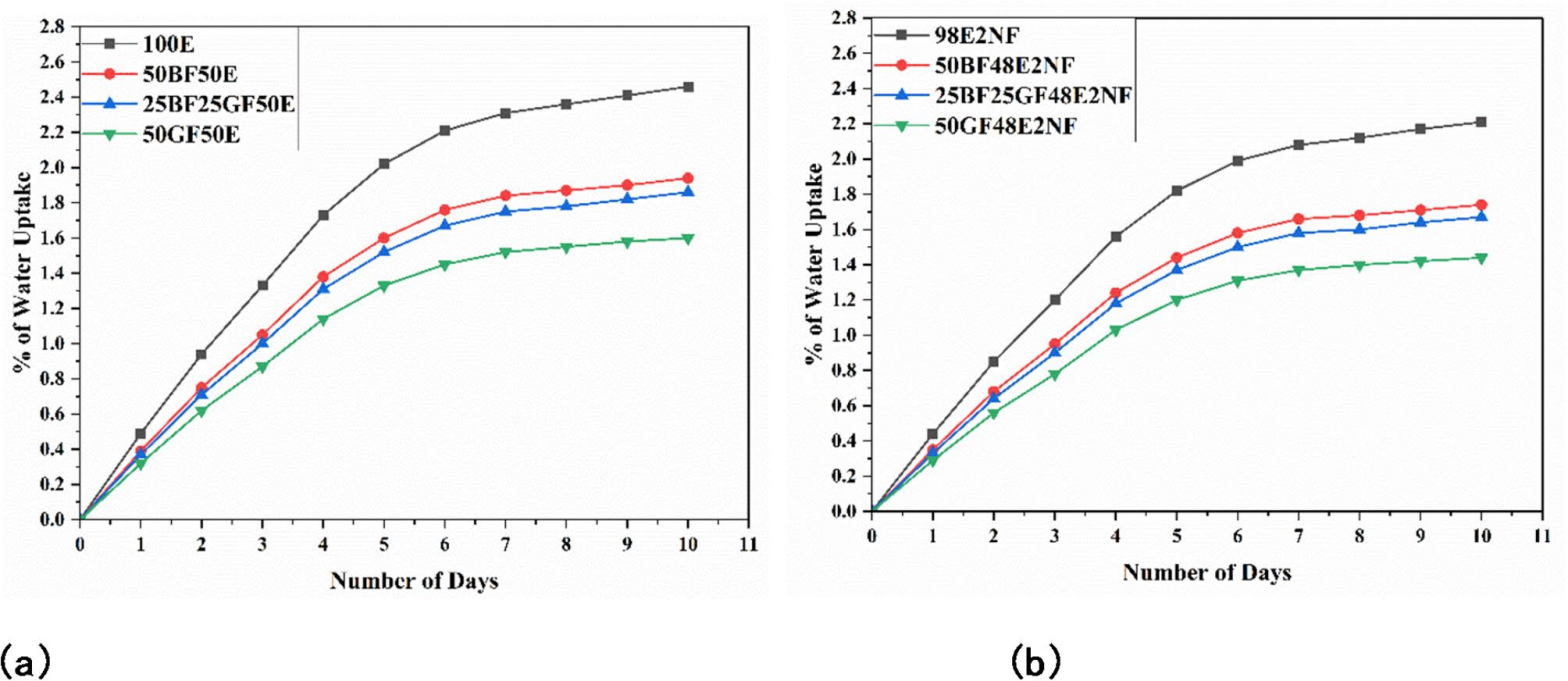
Water uptake curves for process 1 (**a**) without nanoclay (**b**) with nanoclay.

**Fig. 3:**
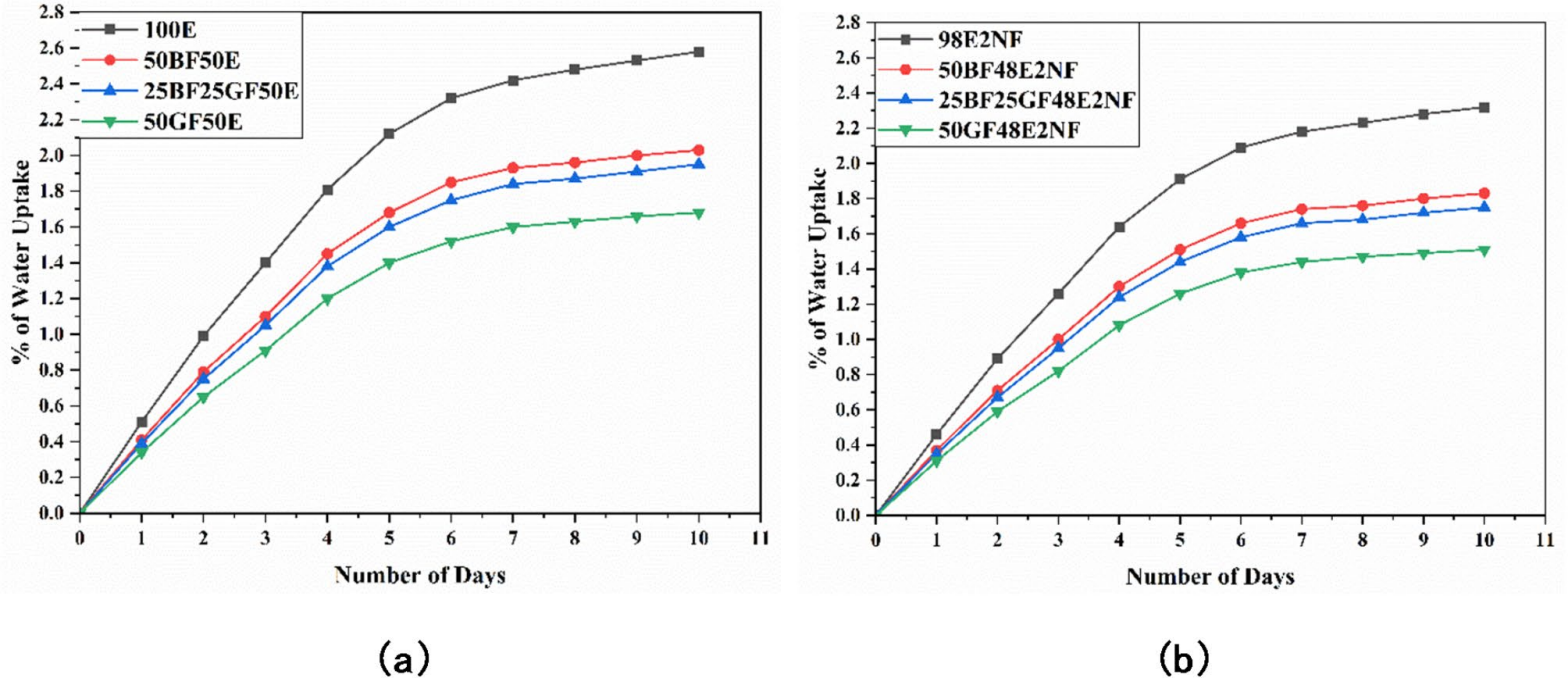
Water uptake curves for process 2 (**a**) without nanoclay (**b**) with nanoclay.

**Fig. 4:**
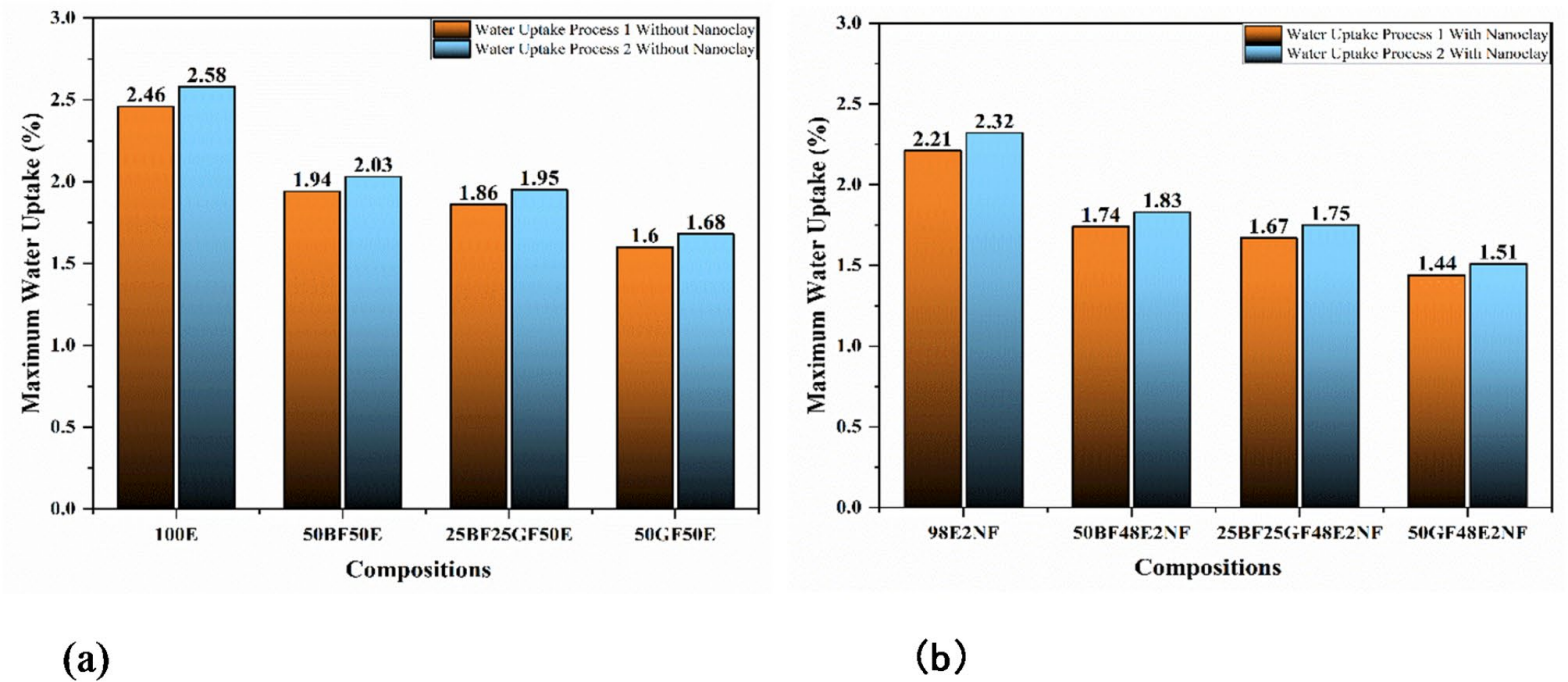
Maximum water uptake (%) (**a**) without nanoclay (**b**) with nanoclay.

Figures 2 and 3 represent graphs that show how the percentage of water uptake corresponds with the period of soaking in boiling water for the specimens. Water uptake is observed in all composites, following a common trend found in polymers. Initially, weight measurements are taken, from which changes are followed over time, enabling the calculation of water uptake percentages. After 10 days, water absorption has become as shown in the figures. Figure 2 shows the water uptake curves of composites over 10 days under process 1, i.e., specimens are immersed in boiling water for 2 h, followed by 22 h of drying (kept outside). Figure 3 shows water uptake curves of composites over 10 days under process 2, i.e., immersed in boiling water for 2 h and retained in the water for 22 h.

As presented in Figs. 2a and 4a, at the end of 10 days under process 1, the water uptake (%) of 100E, 50BF50E, 25BF25GF50E, and 50GF50E are 2.46, 1.94. 1.86 and 1.6%, respectively. Boiling water can accelerate the water uptake into the epoxy resin. This can lead to hydrothermal degradation, where water molecules penetrate the resin matrix and disrupt the polymer cross-links. Also, epoxy resin can absorb water over time, mainly through microvoids^20^. Su et al.^21^ concluded that the water absorption for the pure epoxy resin increases with prolonged immersion time, eventually reaching 2.4 wt% after being immersed for 168 h.

Adding treated bamboo fibers reduces the water uptake (%). The bamboo fibers create a more complex internal structure in the composite, increasing the tortuosity for water molecules. Water must travel around the fibers, making moisture penetrating the matrix harder^9^.

Figure 2a shows that 25BF25GF50E and 50GF50E composites have lesser water uptake (%) than 100E and 50BF50E. Combining treated bamboo and glass fibers can create a hybrid reinforcement system. The glass fibers provide low permeability, while the treated bamboo fibers contribute toughness and flexibility, enhancing the water resistance of the entire composite structure. Also, glass fibers are inherently non-hygroscopic and highly resistant to water uptake. When embedded in the epoxy matrix, they act as a barrier to moisture, preventing water from diffusing through the composite. This creates a physical obstruction, reducing the pathways for water to penetrate the resin^22^.

As presented in Figs. 3a and 4a, at the end of 10 days under process 2, the water uptake (%) of 100E, 50BF50E, 25BF25GF50E, and 50GF50E composites are 2.58, 2.03. 1.95 and 1.68%, respectively. The water uptake (%) of all the composites is higher under process 2; as specimens are retained in the water, there might be a chance of additional mitigation of water molecules.

As shown in Figs. 2b and 3b, and 4b, adding nanoclay reduces the overall water uptake (%) of all the composites under processes 1 and 2. Introducing nanoclay into the epoxy decreases water molecules’ mean free path within the network, reducing water uptake. The greater aspect ratio of nanoclay creates challenging paths for water molecules, significantly contributing to enhanced resistance against water uptake^8,23^.

### Tensile properties of dry and water boiling-soaked specimens

**Fig. 5:**
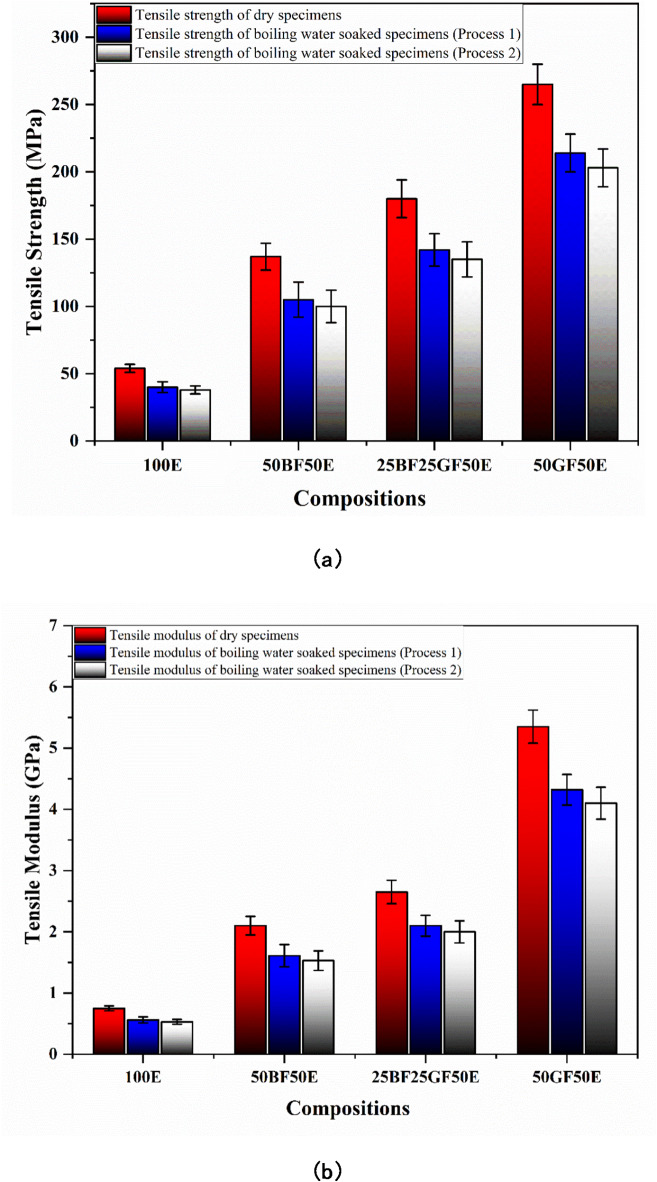
(**a**) Tensile strength and (**b**) Tensile Modulus of dry and boiling water-soaked specimens (without nanoclay).

As shown in Fig. 5a, the tensile strengths of 100E, 50BF50E, 25BF25GF50E, and 50GF50E are 54, 137, 180, and 265 MPa, respectively. Similarly, as shown in Fig. 5b, the tensile modulus of 100E, 50BF50E, 25BF25GF50E, and 50GF50E are 0.75, 2.1, 2.6, and 5 5.35 GPa, respectively. Adding treated bamboo fibers to epoxy resin improves the tensile properties. Bamboo fibers have a naturally high strength-to-weight ratio, which contributes to reinforcing the polymer matrix. The cellulose in bamboo fibers provides strong intermolecular bonding, which enhances the tensile properties when mixed with epoxy resin^24^. When bamboo fibers are incorporated into the epoxy resin, they form a mechanical interlock. This fiber-matrix interaction helps transfer stress from the weaker epoxy matrix to the stronger bamboo fibers, improving overall tensile properties^25^.

25BF25GF50E (hybrid) composite displays improved tensile properties compared to the 50BF50E composite. Bamboo fibers provide inherent strength and flexibility, whereas glass fibers are renowned for their exceptional tensile strength and stiffness. The hybrid composite combines the extraordinary strength of glass fibers with the flexibility of bamboo fibers, resulting in a well-balanced material with improved total tensile properties^19^. The 50GF50E composites exhibit the highest tensile properties compared to all composites and pure epoxy due to glass fibers’ greater strength and stiffness.

As shown in Fig. 5a and b, the tensile strength and modulus of boiling water-soaked specimens decrease compared to dry specimens. For 100E specimens, the tensile strength decreases from 54 MPa to 40 MPa and 38 MPa, and the tensile modulus declines from 0.75 GPa to 0.56 GPa and 0.53 GPa under processes 1 and 2 of boiling water soaking, respectively. This decrease is primarily due to the plasticization of the epoxy matrix as it absorbs water. The absorbed water reduces the cross-linking density of the polymer, leading to a softer, more flexible material with reduced mechanical strength^26^.

As explained earlier, 50BF50E, 25BF25GF50E, and 50GF50E composites have lesser water uptake (%) than 100E, which is the primary reason for the lesser reduction in tensile properties. For the 50BF50E composite, the tensile strength drops from 137 MPa to 105 MPa and 100 MPa, and the tensile modulus declines from 2.1 GPa to 1.61 GPa and 1.53 GPa, under processes 1 and 2 of boiling soaking, respectively. Reinforcing with treated bamboo fibers leads to good fiber-matrix adhesion, creating a rigid interface that limits water ingress. Better adhesion means less moisture can penetrate the fiber-matrix interface, preventing delamination, void formation, or microcracking under boiling water conditions. Adding treated bamboo fibers reduces the amount of water absorbed into the composite, thus limiting the plasticization effect^27^. This helps the composite retain its stiffness and strength, even after being soaked in boiling water.

In the 25BF25GF50E composite, the tensile strength decreases from 180 MPa to 142 MPa and 135 MPa, and the tensile modulus decreases from 2.65 GPa to 2.1 GPa and 2 GPa, under processes 1 and 2 of boiling water soaking, respectively. When treated bamboo fibers are combined with glass fibers in a hybrid composite, they can synergistically improve the mechanical properties while minimizing the water uptake^9^. The treated bamboo fibers add toughness and flexibility, while the glass fibers add stiffness and strength. This hybrid approach helps maintain the mechanical performance of the composite even under boiling water conditions.

The 50GF50E composite shows the highest tensile strength, reducing from 265 MPa to 214 MPa and 203 MPa under processes 1 and 2 of boiling soaking, respectively. Similarly, the 50GF50E composite shows the highest tensile modulus, declining from 5.35 GPa to 4.32 GPa and 4.1 GPa under processes 1 and 2 of boiling soaking. This is because glass fibers are highly resistant to water absorption and maintain their mechanical properties even after exposure to water. The reduction in strength is mainly due to water absorption by the epoxy matrix rather than the fibers themselves. Additionally, the glass fibers provide superior mechanical reinforcement due to their high strength and stiffness, contributing to the overall strength of the composite^28^.

**Fig. 6:**
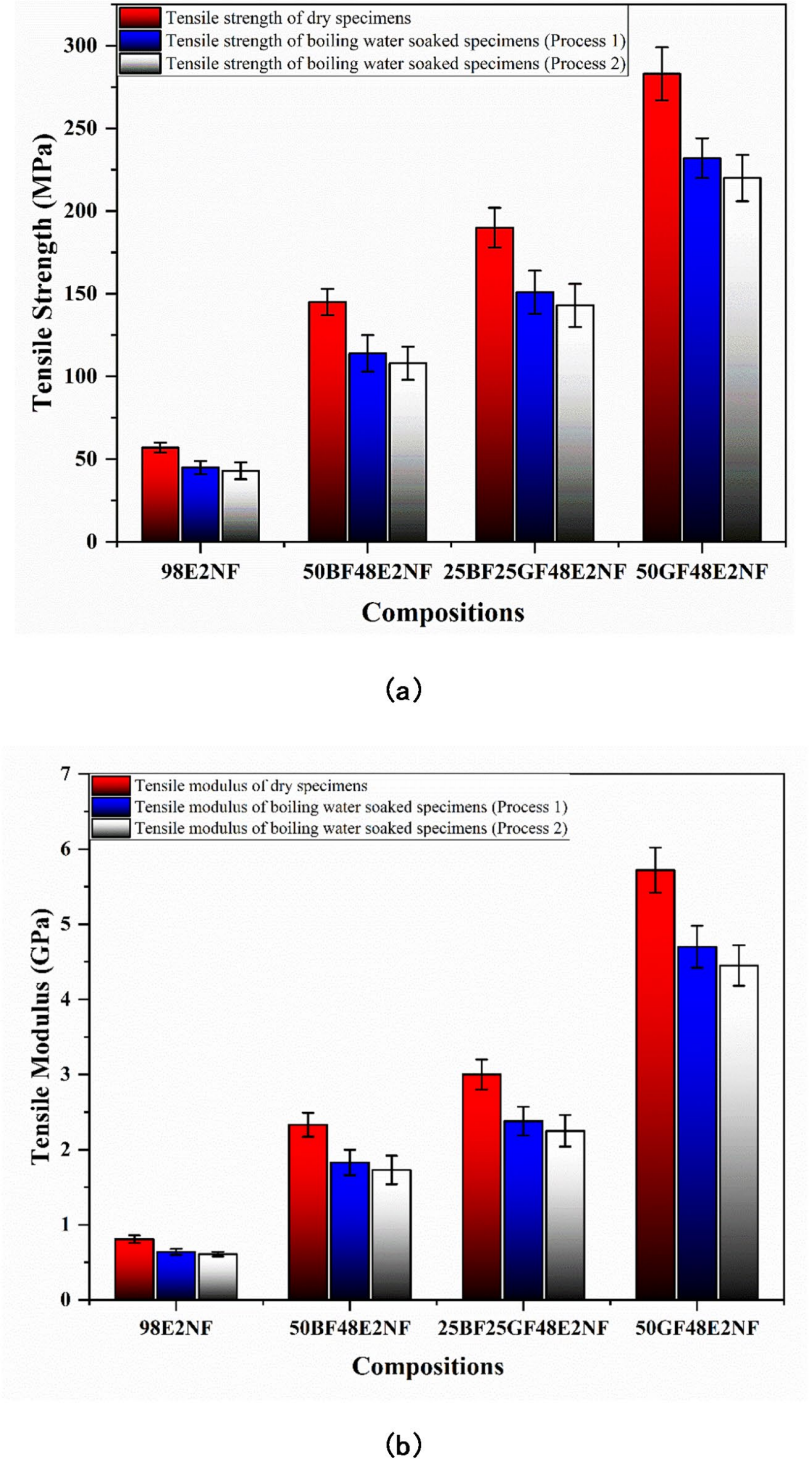
(**a**) Tensile strength and (**b**) Tensile Modulus of dry and boiling water-soaked specimens (with nanoclay).

As presented in Fig. 6a and b, nanoclay enhances the tensile strength and modulus of the epoxy and all the composites. The tensile strengths of 98E2NF, 50BF48E2NF, 25BF25GF48E2NF, and 50GF48E2NF composites are 57, 145, 190, and 283 MPa, respectively, which are much higher than the epoxy and composites without nanoclay (as presented in Fig. 5a). As displayed in Fig. 6 (b), the tensile modulus of 98E2NF, 50BF48E2NF, 25BF25GF48E2NF, and 50GF48E2NF composites are 0.81, 2.33, 3, and 5.72 GPa, respectively, which are much higher than the epoxy and composites without nanoclay (as presented in Fig. 5b).

Many researchers have reported that incorporating nanoclay can enhance epoxy and composites’ tensile strength and modulus through various mechanisms: (1) Nanoclay particles, with their high surface area, increase the interaction between the matrix and the reinforcing fibers. This leads to stronger interfacial adhesion, which helps transfer stress more effectively across the matrix and fibers during loading^29,30^. (2) The layered structure of nanoclay acts as a barrier to crack propagation. This hinders the growth of microcracks within the composite material, making it more resistant to failure under tensile loads^31^. (3) The addition of nanoclay increases the stiffness of the polymer matrix, thereby reducing its deformation under stress. This enhanced stiffness contributes to an overall increase in tensile properties^32^.

Also, the addition of nanoclay reduces the boiling water-soaking effect, which declines the percentage of reduction in tensile properties in all the specimens. Adding nanoclay to epoxy (98E2NF) reduces the tensile strength by 21 and 25% compared to dry specimens under processes 1 and 2 of boiling soaking, respectively, which are much less than pure epoxy (100E) specimens, i.e., 26 and 29% reduction in tensile strength. Meanwhile, the tensile modulus of 98E2NF decreases by 20 and 24% compared to dry specimens under processes 1 and 2 of boiling soaking, respectively.

Similarly, adding the nanoclay reduces the percentage of reduction in tensile strength of composites, ranging from 18 to 21% and 22 to 24% compared to dry specimens under processes 1 and 2 of boiling water soaking, respectively, which are much less than the composites without nanoclay, i.e., 19 to 24% and 23 to 27% reduction in tensile strength. The percentage tensile modulus of composites reduces from 17 to 22% compared to dry specimens under process 1 of boiling water soaking and from 22 to 26% compared to dry specimens under process 2 of boiling water soaking. Adding nanoclay can mitigate the detrimental effects of boiling water soaking on tensile properties by acting as a barrier to water diffusion, improving interfacial adhesion, imparting hydrophobicity, and reducing matrix swelling^33^.

The tensile properties of all the composite specimens under boiling water soaking process 2 declines more than process 1. As presented in Fig. 4a, the water uptake (%) of all the composites is higher under process 2; as specimens are retained in the water, there is an additional mitigation of water molecules. The additional mitigated water molecules affect the tensile properties more.

### SEM analysis of dry and boiling water-soaked specimens

**Fig. 7:**
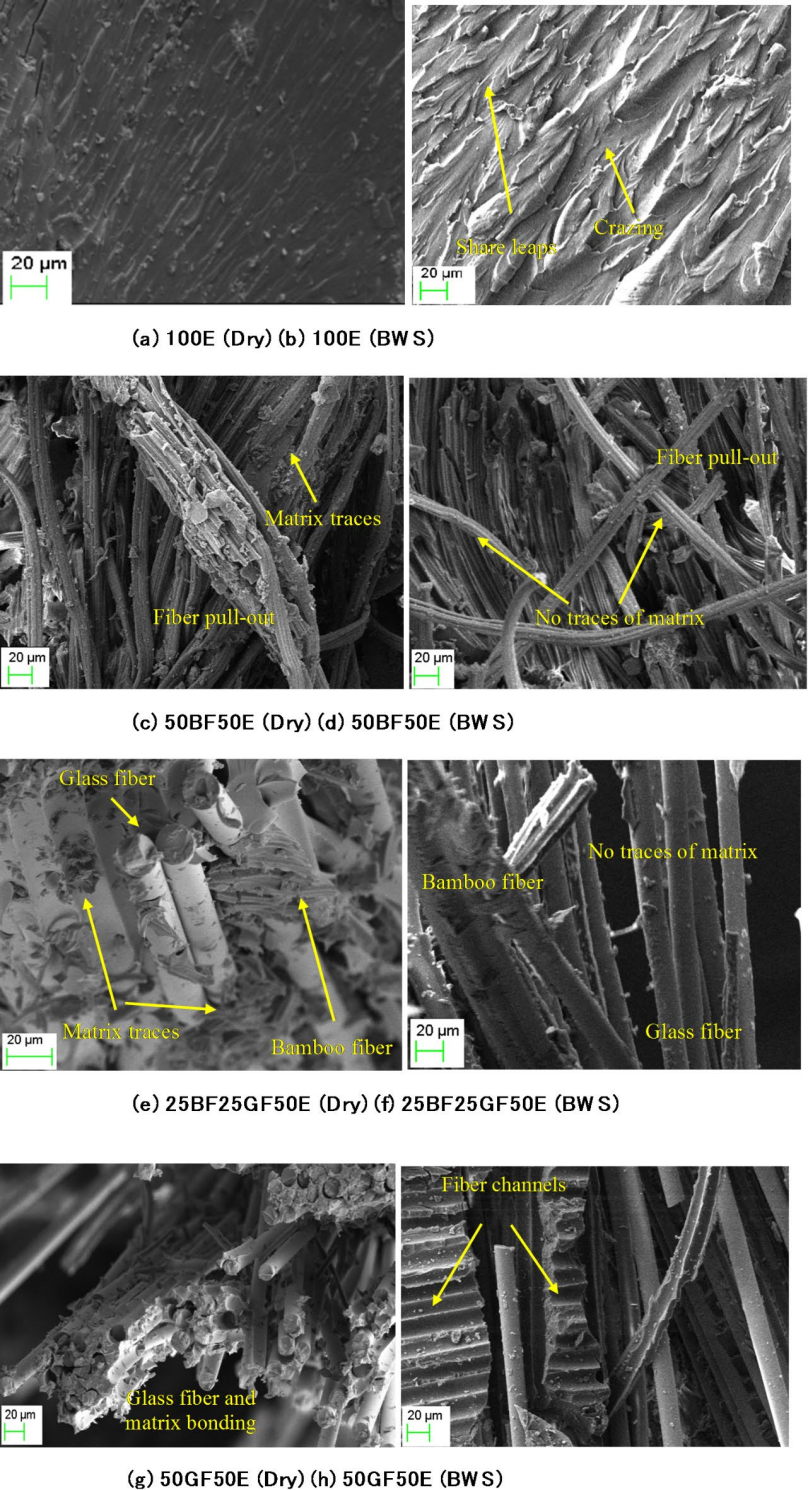
SEM analysis of dry and boiling water-soaked specimens (Without nanoclay).

Figure 7 presents SEM images of dry and boiling water-soaked specimens, highlighting noticeable differences between the two conditions. Figure 7a, b presents the SEM images of dry and boiling water-soaked specimens of 100E. In Fig. 7a, the 100E (pure epoxy) specimen exhibits a relatively smooth fracture surface morphology. In contrast, the boiling water-soaked 100E specimen in Fig. 7b shows signs of phase separation, degradation (shear leaps and crazing), or leaching of components caused by water infiltration.

Figure 7c–h illustrates the failure of specimens under tensile load characterized by fiber pull-out, matrix rupture, and fiber breakage. The fibers are encased in a significant amount of matrix residues, suggesting a robust bond between the fibers and the matrix. The interfacial adhesion is so strong that cracks form around the pulled fibers, as seen in Fig. 7c, e, g.

Specimen failure under boiling water-soaked conditions (Fig. 7d, f, h) is caused by matrix and fiber-matrix interface degradation, including matrix cracking or rupture. Figure 7d, f, h reveal the absence of matrix material on the fibers, indicating how boiling water soaking deteriorates the fiber-matrix interface. This degradation explains the reduced tensile properties of boiling water-soaked composites compared to dry composites.

**Fig. 8:**
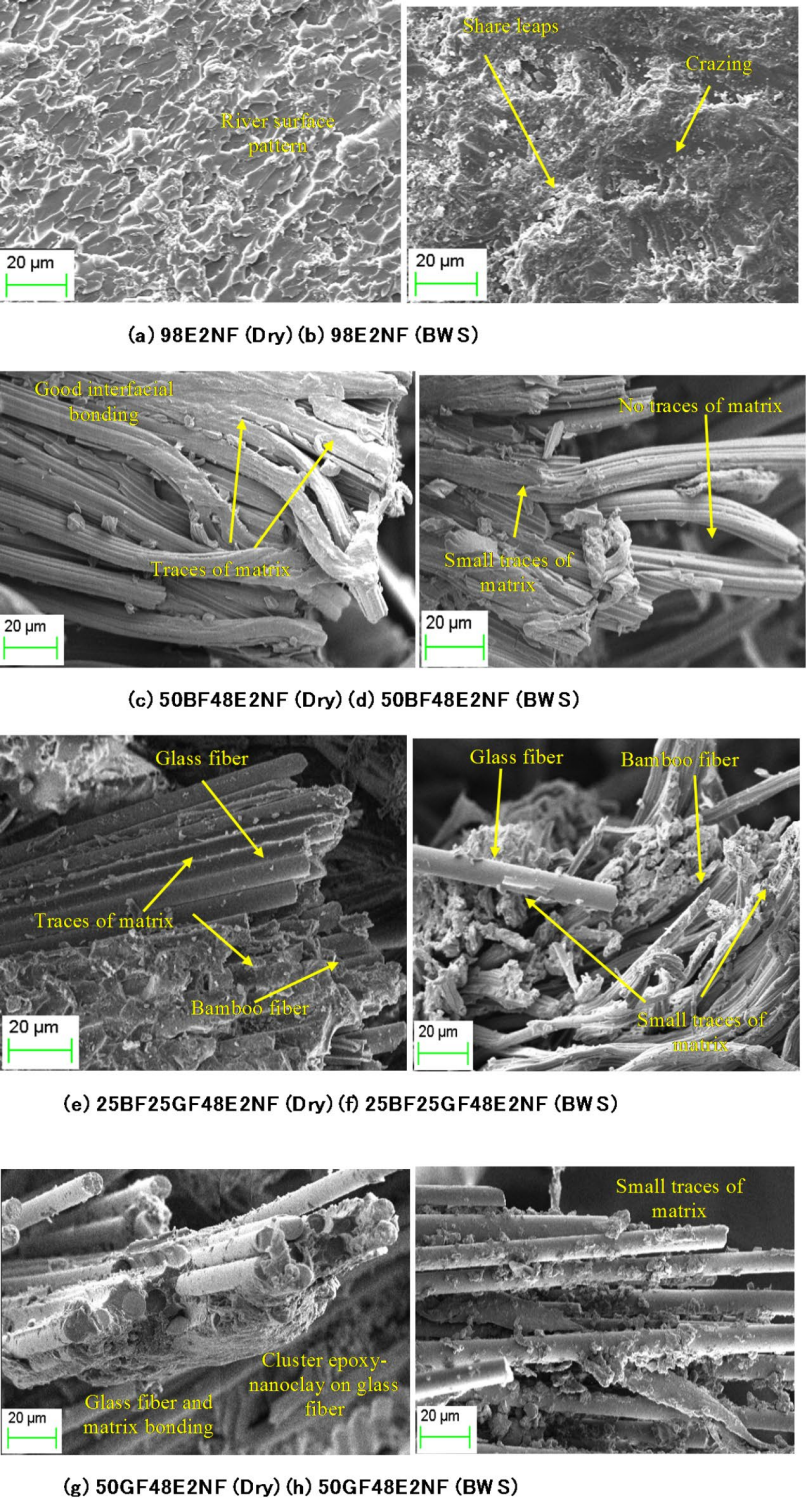
SEM analysis of dry and boiling water-soaked specimens (With nanoclay).

Figure 8 presents the SEM images of dry and boiling water-soaked specimens with nanoclay. The fracture surfaces of the 100E specimen (Fig. 7a) and the 98E2NF specimen (Fig. 8a) reveal notable differences. The 98E2NF specimen exhibits a more intricate morphology, with nanoclay producing a river surface-like texture. Nanoclay is an effective reinforcement, enhancing tensile strength by improving load transfer and limiting crack propagation within the epoxy matrix. Compared to the dry 98E2NF specimen (Fig. 8a), the boiling water-soaked 98E2NF specimen (Fig. 8b) shows a smoother surface with shear leaps and crazing. The boiling water-soaked 100E specimen (Fig. 7b) is more affected by boiling water-soaking conditions than the boiling water-soaked 98E2NF specimen (Fig. 8b). Nanoclay forms barriers for water molecules to penetrate into the epoxy resin, significantly improving resistance to water absorption. This likely explains the slight reduction in tensile properties witnessed in 98E2NF specimens compared to 100E specimens.

Figure 8c–h illustrates that adding nanoclay has strengthened the interfacial bonding between the fibers and the matrix, improving the tensile properties of the composites. When comparing the SEM images of the 50BF50E, 25BF25GF50E, and 50GF50E composites (Fig. 7c–h) with those of the 50BF48E2NF, 25BF25GF48E2NF, and 50GF48E2NF composites (Fig. 8c–h), the latter reveals larger clusters of matrix attached to the fibers. This suggests enhanced fiber-matrix bonding, which could explain the increased tensile properties observed in the 50BF48E2NF, 25BF25GF48E2NF, and 50GF48E2NF composites.

Boiling water-soaking negatively impacts the composites. In comparison to dry specimens (Fig. 8c, e, g), the boiling water-soaked specimens (Fig. 8d, f, h) show smaller matrix clusters on the fibers, indicating degradation of both the matrix and the fiber-matrix interface. However, the boiling water-soaked specimens of the 50BF48E2NF, 25BF25GF48E2NF, and 50GF48E2NF composites (Fig. 8d, f, h) display more matrix clusters on the fibers than the 50BF50E, 25BF25GF50E, and 50GF50E composites (Fig. 7d, f, h). This suggests that including nanoclay has reduced the adverse effects of water-soaking. As a result, there is a minor reduction in tensile strength for the 50BF48E2NF, 25BF25GF48E2NF, and 50GF48E2NF composites compared to the 50BF50E, 25BF25GF50E, and 50GF50E composites.

### Flexural properties of dry and water boiling-soaked specimens

**Fig. 9:**
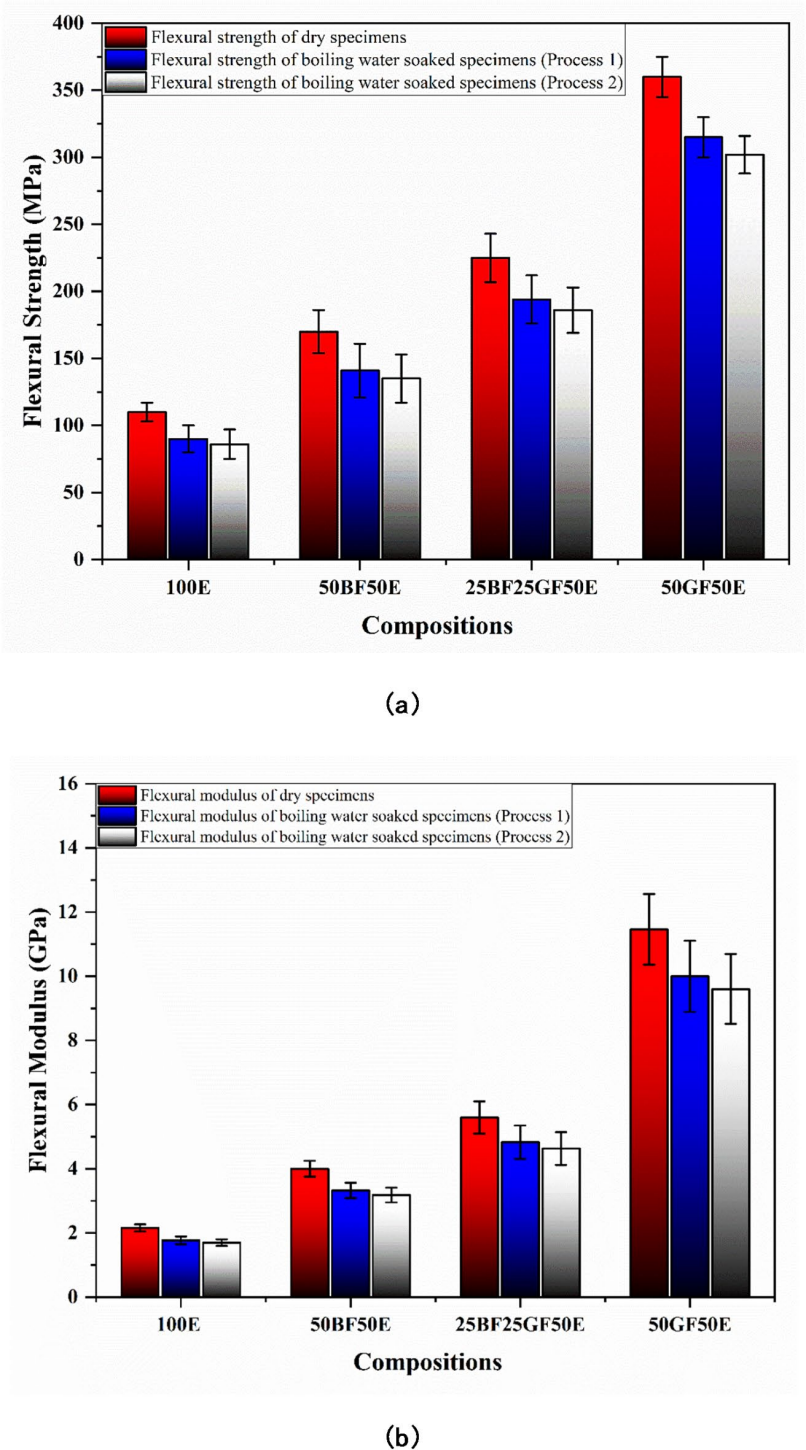
(**a**) Flexural strength and (**b**) Flexural modulus of dry and boiling water-soaked specimens (without nanoclay).

As shown in Fig. 9a, the flexural strengths of 100E, 50BF50E, 25BF25GF50E, and 50GF50E are 110, 170, 225, and 360 MPa, respectively. Meanwhile, as displayed in Fig. 9b, the flexural modulus of 100E, 50BF50E, 25BF25GF50E, and 50GF50E are 2.16, 4, 5.6, and 11.46 GPa, respectively. Compared to pure epoxy (100E), bamboo and glass fiber-reinforced epoxy composites exhibit higher flexural properties because the fibers have higher mechanical strength than the epoxy resin, and their inclusion improves the load-bearing ability, allowing the composite to withstand greater flexural forces^34^. The fibers transfer applied stress more efficiently through the interface between the fibers and the epoxy. This interaction enhances the stiffness and strength of the composite, especially under flexural loading^35^.

Figure 9a shows that the flexural strength of boiling water-soaked specimens decreases compared to dry specimens. For 100E specimens, flexural properties decrease by 18% and 22% under processes 1 and 2 of boiling soaking, respectively. The presence of water in the 100E breaks down the chemical bonds and reduces the material’s overall strength^36^. Like tensile strength, the flexural strength of boiling water-soaked 50BF50E, 25BF25GF50E, and 50GF50E composites decreases by 17, 14, and 12.5% under process 1 of boiling soaking and 20, 17 and 16% under process 2 of boiling soaking, respectively. Meanwhile, the flexural modulus of boiling water-soaked 50BF50E, 25BF25GF50E, and 50GF50E composites decreases by 18, 15, and 13% under process 1 of boiling soaking and 21, 18 and 17% under process 2 of boiling soaking, respectively.

The reduction of flexural properties of composite specimens exposed to boiling water soaking can be attributed to two reasons. The first, engrossed water can lead to degradation at the interface of fibers and the matrix material, causing diminished adhesion and bonding between the fibers and matrix. The second is the presence of water in the composite structure, which causes osmotic cracking and reduces adhesion. Finally, the decrease in adhesion results in a decline in flexural properties^37,38^. The reduction in flexural strength and modulus of composite samples subjected to hydrothermal aging can also be explained by the fact that the fiber swelling increased the water gain and degraded the interface between the fiber and matrix, resulting in microvoids between the polymer chains and cracking^39,40^.

Flexural strength decreases due to the water molecules filling the spaces between polymer chains and speeding up matrix breaking. The polymer chains generally become more mobile, and their flexural properties diminish due to the plasticizing effect of water molecules^41^.

**Fig. 10:**
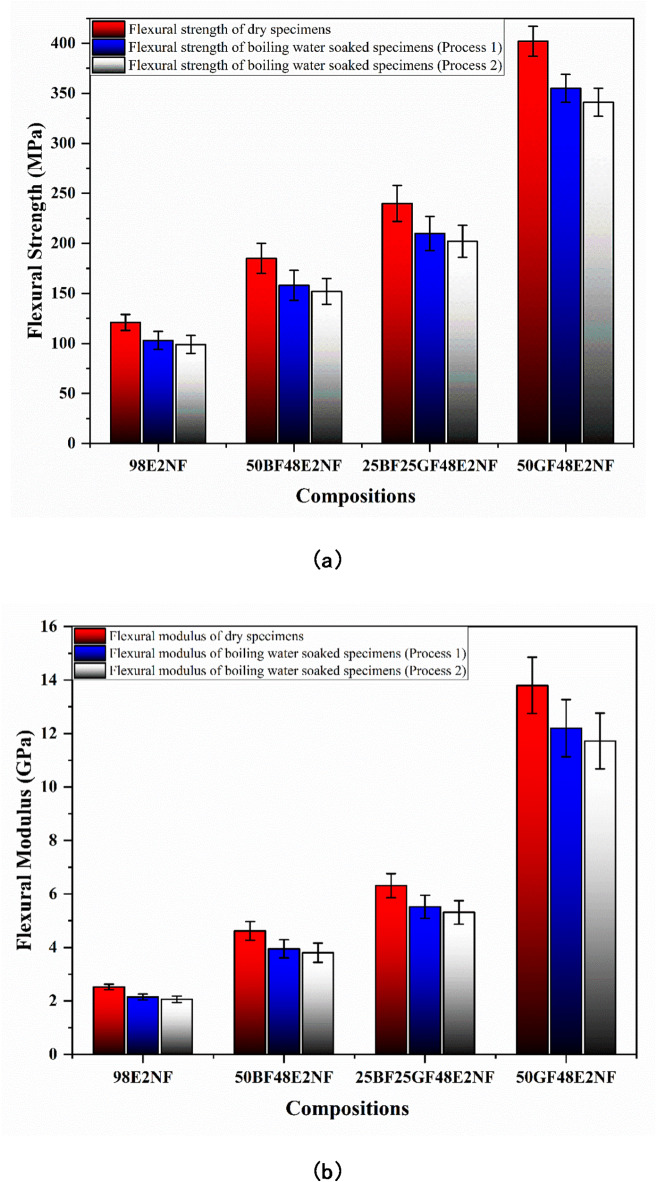
(**a**) Flexural strength and (**b**) Flexural modulus of dry and boiling water-soaked specimens (with nanoclay).

As presented in Fig. 10a, nanoclay enhances the flexural strength of the epoxy and all the composites. The flexural strengths of 98E2NF, 50BF48E2NF, 25BF25GF48E2NF, and 50GF48E2NF composites are 121, 185, 240, and 402 MPa, respectively, which are much higher than the epoxy and composites without nanoclay (as presented in Fig. 9a). As displayed in Fig. 10b, the flexural modulus of 98E2NF, 50BF48E2NF, 25BF25GF48E2NF, and 50GF48E2NF composites are 2.52, 4.62, 6.31, and 13.8 GPa, respectively, which are much higher than the epoxy and composites without nanoclay (as presented in Fig. 9b). Nanoclay particles help to enhance the interaction between the fibers and the polymer matrix. This stronger interfacial bonding allows for better load transfer between the fibers and the matrix, increasing the composite’s ability to resist bending and deformation under load^42^.

Also, adding nanoclay reduces the boiling water-soaking effect, which declines the percentage of reduction in flexural strength in all the specimens. Adding nanoclay to epoxy (98E2NF) reduces the flexural properties by 14 and 19% compared to dry specimens under processes 1 and 2 of boiling soaking, respectively, which are much less than pure epoxy (100E) specimens.

Similarly, adding the nanoclay reduces the percentage of reduction in flexural strength of composites, ranging from 12 to 14% and 15 to 17% compared to dry specimens under processes 1 and 2 of boiling water soaking, respectively, which are much less than the composites without nanoclay. Also, adding the nanoclay reduces the percentage of reduction in flexural modulus of composites, ranging from 11 to 15% and 15 to 18% compared to dry specimens under processes 1 and 2 of boiling water soaking, respectively. Nanoclay reduces the moisture absorption of the composite. Lower moisture absorption can help prevent the polymer matrix’s degradation and the composite’s weakening over time^43^. Also, nanoclay prevents or minimizes the formation of microcracks, preserving the structural integrity of the composite.

Like the tensile properties, the flexural properties of all the composite specimens under boiling water soaking process 2 declines more than process 1. As presented in Fig. 4a, the water uptake (%) of all the composites is higher under process 2; as specimens are retained in the water, there is an additional mitigation of water molecules. The additional mitigated water molecules affect the flexural properties more.

## Conclusions

1. All composites’ maximum water uptake (%) is 1.6 to 2.46% under boiling water soaking process 1 and 1.68 to 2.58% under boiling water soaking process 2. The addition of nanoclay declines the water uptake (%), ranging from 1.44 to 2.21% under boiling water soaking process 1 and 1.51 to 2.32% under boiling water soaking process 2.
2. Reinforcing bamboo and glass fibers with epoxy resin increases tensile and flexural properties. The 50BF50E displays the tensile strength, tensile modulus, flexural strength, and flexural modulus of 137 MPa, 2.1 GPa, 170 MPa, and 4 GPa, respectively. Meanwhile, the 50GF50E displays the tensile strength, tensile modulus, flexural strength, and flexural modulus of 265 MPa, 5.32 GPa, 360 MPa, and 11.46 GPa, respectively.
3. A comparison between dry and boiling water-soaked specimens shows that boiling water exposure negatively impacts all composites, resulting in lower tensile and flexural properties than the dry specimens.
4. For 100E specimens, the tensile properties decrease by 25 and 30%, and the flexural properties decline by 18 and 22% under processes 1 and 2 of boiling soaking, respectively. Meanwhile, for fiber-reinforced composites, the tensile properties decrease by 19 and 27%, and the flexural properties decline by 12 and 20% under processes 1 and 2 of boiling soaking, respectively.
5. Adding nanoclay enhances composites’ tensile and flexural properties by 5 to 7% and 10 to 12%, respectively. Also, nanoclay declines the effect of boiling water soaking on the properties of composites.
6. For 98E2NF specimens, the tensile properties decrease by 20 and 25%, and the flexural properties decline by 14 and 19% under processes 1 and 2 of boiling soaking, respectively. Meanwhile, for fiber-reinforced composites, the addition of nanoclay decreases the tensile properties by 17 and 26%, and the flexural properties decline by 11 and 18% under processes 1 and 2 of boiling soaking, respectively.
7. The causes of specimen failure under tensile load are revealed by SEM analysis of the fracture surfaces, which shows evident variations between dry specimens and those soaked in boiling water.

The assessment of material properties after boiling water soaking is crucial for evaluating the durability and performance of materials under extreme moisture and thermal conditions, simulating real-world environments such as marine, automotive, and construction applications. Boiling water soaking tests accelerate water absorption and hydrothermal aging effects, revealing degradation mechanisms like fiber-matrix debonding, which impact mechanical integrity.

## Data Availability

The data used and/or analysed during the current study available from the corresponding author on reasonable request.
